# Phosphoproteomic Analysis of Cell-Based Resistance to BRAF Inhibitor Therapy in Melanoma

**DOI:** 10.3389/fonc.2015.00095

**Published:** 2015-05-15

**Authors:** Robert Parker, Laura J. Vella, Dylan Xavier, Ardeshir Amirkhani, Jimmy Parker, Jonathan Cebon, Mark P. Molloy

**Affiliations:** ^1^Australian Proteome Analysis Facility, Department of Chemistry and Biomolecular Sciences, Macquarie University, Sydney, NSW, Australia; ^2^Cancer Immunology Group, Olivia Newton-John Cancer Research Institute, Ludwig Institute for Cancer Research, School of Cancer Medicine, La Trobe University, Heidelberg, VIC, Australia; ^3^NHS Trust Southport and Ormskirk General Hospital, Ormskirk, UK

**Keywords:** phosphoproteomics, BRAF, drug resistance, vemurafenib, kinases, label-free quantitation, mass spectrometry

## Abstract

The treatment of melanoma by targeted inhibition of the mutated kinase BRAF with small molecules only temporarily suppresses metastatic disease. In the face of chemical inhibition tumor plasticity, both innate and adaptive, promotes survival through the biochemical and genetic reconfiguration of cellular pathways that can engage proliferative and migratory systems. To investigate this process, high-resolution mass spectrometry was used to characterize the phosphoproteome of this transition *in vitro*. A simple and accurate, label-free quantitative method was used to localize and quantitate thousands of phosphorylation events. We also correlated changes in the phosphoproteome with the proteome to more accurately determine changes in the activity of regulatory kinases determined by kinase landscape profiling. The abundance of phosphopeptides with sites that function in cytoskeletal regulation, GTP/GDP exchange, protein kinase C, IGF signaling, and melanosome maturation were highly divergent after transition to a drug resistant phenotype.

## Introduction

In melanoma, coding mutations in the mitogen-activated kinase pathway (MAPK) (e.g., BRAF and RAS) are common and contribute to disease severity (1). In cutaneous melanoma, BRAF is mutated in ~70% of cases and correlates with poorer prognosis and aggressive disease (2, 3). The mutant BRAF protein is a hyperactive serine/threonine-protein kinase that directs signaling through MEK1/2 to phosphorylate the MAPK ERK1/2 and drive cell proliferation and tumor growth. In recent years, a high-therapeutic value has been attained by targeted inhibition of the mutated BRAF protein with selective inhibitors (e.g., vemurafenib and dabrafenib) (4–6). Vemurafenib and dabrafenib effectively reduce signaling through the MAPK pathway, resulting in disease regression (~85%) and progression free survival for ~5–6 months [reviewed in Ref. (7)], after which almost all treated patients develop drug resistant, progressive disease (5).

Several mechanisms for intrinsic and acquired resistance have been detected *in vivo* and *in vitro* and this has been extensively reviewed (7–9). Relapses in melanoma involve mechanisms that reprogram signaling pathways to bypass inhibition and reactivate the ERK1/2 signaling hub (10). For example, the up-regulation of receptor tyrosine kinases (RTKs), platelet derived growth factor (PDGF), epidermal growth factor receptor (EGFR), and insulin-like growth factor 1 receptor (IGF-1R) can drive cell survival signals through the PI3K/AKT pathway (11–13). Alternative pathways that reactivate ERK during targeted therapy utilize the multimeric properties of RAF signaling complexes and also occur in BRAF wild-type cells. BRAF inhibitors have been shown to drive the formation of alternative RAF dimers able to phosphorylate MEK and induce ERK signaling (14–17). In drug resistant patients, up-regulation and splicing of MAPK signaling components [CRAF, BRAF, or the MAP3K8 (COT)] provide alternative mechanisms for the reactivation of ERK1/2 signaling (18–20). In response to the microenvironment, phenotypic switching can also occur based upon intrinsic tumor heterogeneity and lead to resistance to therapy (21). For example, paracrine signaling from stromal cells that secrete hepatocyte growth factor (HGF) reestablish the MAPK pathway in BRAF mutated cells by activating the RTK MET (22). Independent of the MAPK pathway, low expression of the melanocyte transcriptional network driver microphthalmia-associated transcription factor (MITF) associates with drug resistance and a more invasive, less proliferative phenotype (23, 24). This and the fact that MAPK inhibitors can themselves drive an invasive phenotype (25) indicate that inter-tumor plasticity allows melanoma to evade complete growth arrest in the face of MAPK inhibition.

The discovery of these mechanisms and others [reviewed in Ref. (8)] has established opportunities for novel melanoma treatment. The design of more effective co-inhibitory-based therapies could represent an improved strategy to prevent the acquired resistant phenotypes currently found in the clinic. In most cases, combination therapies in which BRAF inhibition is paired with inhibitors of the well establish mediators of resistance (PI3K, MEK, HGF, and IGF-R1) is showing promise (12, 26, 27). Because kinases (ERK1/2, IGF-R1, MEK, PI3K) provide key signaling hubs that orchestrate biochemical processes in drug resistant melanoma, characterizing their global activity profiles will aid the design of novel therapies. Kinase activity can be mapped by measuring the abundance of substrates using phosphoproteomic methods that combine phosphopeptide enrichment with high-resolution mass spectrometry (28). A quantitative phospho-site (P-site) analysis has the potential to provide a direct readout of kinase activity, elucidate novel mechanisms driving resistance, and guide the selection of therapies for validation in cell and animal models (29, 30). Previously, Old et al. reported ~90 P-sites that were regulated in a melanoma cell line in response to short-term MKK1/2 inhibition and Girotti et al. screened the phosphoproteome of A375 cells in a model for *in vitro* acquired drug resistance (31, 32). Both studies identify changes in the intensity of P-sites in signaling and cytoskeletal regulators and support the co-inhibition of specific kinase signaling (EGFR-SRC and SFK-STAT3) to provide therapeutic efficacy in drug resistance (32). To add to this work, we have developed and applied a simple and reproducible label-free quantitative phosphoproteomic procedure and analyzed an *in vitro* model of acquired drug resistance in melanoma cell line LM-MEL-28. The abundance of 2230 P-sites was measured by high-resolution mass spectrometry and correlated with the abundance of 3556 unmodified proteins to provide a more accurate determination of kinase activity. Kinase-substrate databases (Phosphosite.org, cell signaling) and motif analysis (NetworKIN) of the flanking linear amino acid sequences predicted several regulatory kinases that are most likely to be responsible for differential phosphorylation detected during long-term exposure to BRAF inhibition in LM-MEL-28. Key regulatory sites that control actin and microtubule-based cytoskeleton and cellular GTP/GDP ratio exhibited large changes in phosphorylation. Phosphorylation of the melanosome G-protein coupled receptor (GPCR) OA1 (GP143) indicated a direct role for the melanocyte maturation pathway. While sites of phosphorylation of the insulin receptor substrate IRS-2 and IGFR2 indicated novel points of regulation in the IGF-1R pathway previously identified to mediate drug resistance in melanoma.

## Materials and Methods

### Cell culture and protein preparation

The melanoma cell line LM-MEL-28 was selected from the Ludwig Institute for Cancer Research Melbourne Melanoma Cell Line Panel (33). LM-MEL-28 was cultured in RPMI 1640 medium supplemented with 10% (v/v) bovine serum (Life Technologies) at 37°C in a humidified atmosphere of 5% CO_2_. Cells were treated with PLX4720 (Selleck Chemicals) for a 1-month period in 5 μM PLX4720 to generate a drug resistant line referred to LM-MEL-28R1. Cells were tested for authenticity by short tandem repeat profiling according to the ANSI/ATCC ASN-0002-2011 standards. For phosphoproteomic analysis, six biological replicates were generated by sub-culture and cells were grown to 80–90% confluence with the LM-MEL-28-R1 continuously cultured in the presence of 5 μM PLX4720 and LM-MEL-28 in the presence of vehicle. Cells were washed three times in PBS and harvested by gentle enzyme-based release (TrypLE), pooled and centrifuged at 400 × *g*, cell pellets frozen on dry ice and stored at −70°C. Cells were lysed by boiling for 5 min in 1% (w/v) sodium deoxycholate (Sigma), 50 mM triethylammonium bicarbonate (TEAB) (Sigma), and 1 mM MgCl_2_ (Sigma). Lysates were cooled to 4°C, sonicated to complete lysis, and DNA was digested by incubation with benzonase (Sigma) (10,000 units). Lysate were centrifuged at 20,000 × *g* for 10 min and protein amounts determined by the micro-BCA assay (Pierce). Samples were stored at −80°C. Mutational testing was performed by MelCarta assay and all cell lines were tested for mycoplasma and appropriate consent from all patients had been obtained.

### Protein digestion and phosphopeptide enrichment

Five hundred milligrams of total protein lysate were reduced with 5 mM DTT (Sigma) for 30 min at 60°C and alkylated with 10 mM of iodoacetamide (Sigma) in the dark at room temperature for 30 min. Trypsin (Promega) was added at ratio of 1:50 ratio for 18 h at 37°C. Samples were adjusted to 1% (v/v) trifluoroacetic acid (TFA) (Sigma), 80 mg/ml glycolic acid (Sigma), and the precipitated deoxycholate was removed by centrifugation. Five milligrams of TiO_2_ beads (Titanisphere, 10 μm) were washed once in 0.1% (v/v) TFA, 70% (v/v) acetonitrile (ACN), and 80 mg/ml glycolic acid, added directly to the sample and incubated with shaking for 1 h. A C8 stage-tip was prepared and washed with methanol (Sigma), then 0.1% (v/v) TFA, 70% (v/v) ACN, and 80 mg/ml glycolic acid (40 μl). TiO_2_ beads were added to the C8 stage-tip and tips were centrifuged 1000 × *g* until all liquid was dispensed. Beads were washed on tip with 300 μl of 0.1% TFA, 70% ACN, 80 mg/ml glycolic acid (300 μl) twice then thrice with 0.1% (v/v) TFA and 70% (v/v) ACN. Phosphopeptides were eluted from TiO_2_ tip with consecutive 100 μl additions of 1% (v/v) ammonia (Sigma) with 0, 30, and 50% (v/v) ACN. Samples were immediately dried and resuspended in 1% (v/v) TFA and 5% (v/v) ACN for LC-MS/MS.

### Isobaric labeling by reductive dimethylation and peptide separation

Proteolytic digestion of 100 μg total protein was carried out as described above and samples were labeled by reductive dimethylation using formaldehyde isotopologues (34) with slight modifications (35). After labeling, each sample was pooled and 40 μg separated into six fractions using pH-based strong anion exchange (SAX) STAGE tips (36) described in Ref. (37).

### Mass spectrometry (LC-MS/MS)

Samples were loaded onto a self-packed 100 μm × 3.5 cm reversed phase peptide trap (Solid core Halo^®^ 2.7 μm 160 Å ES-C18, Advanced Materials Technology) and desalted for 10 min with buffer A [0.1% (v/v) formic acid], peptide separation was carried out using a self-packed 75 μm × 10 cm (Solid core Halo^®^ 2.7 μm 160 Å ES-C18, Advanced Materials Technology) column. A buffer B [100% (v/v) ACN, 0.1% (v/v) formic acid] gradient (5–40% in 120 min) was used to elute peptides. Phosphopeptides were ionized by electrospray ionization and data-dependent MS/MS acquisition carried out using a Q-Exactive consisting of 1 full MS1 (*R* = 70 K) scan acquisition from 350 to 1500 *m*/*z*, and 10 HCD type MS2 scans (*R* = 15 K). Dimethylated peptides were analyzed on an Orbitrap Elite (Thermo Fisher Scientific) consisting of 1 full MS^1^ (*R* = 120 K) scan acquired from 350 to 1500 *m*/*z*, and 10 CID type MS^2^ scans. On both instruments, monoisotopic precursor selection, charge state screening, and dynamic exclusion were enabled, charge states of +1, >4, and unassigned charge states were not subjected to MS^2^ fragmentation. Raw mass spectra were identified using Maxquant 1.3 using a 1% peptide and protein FDR. Searches were conducted against the uniprot complete proteome reference database downloaded on June 06, 2014. The database was supplemented with common contaminants often found in cell culture and proteomics experiments these were later removed. Searches specified for tryptic peptides with four missed cleavages, 7 ppm precursor ion mass tolerance, 0.05 Da fragment ion mass tolerance, fixed modifications of carbamidomethylation (C), and variable modification of oxidation (M), acetylation (N-term, protein), and phosphorylation (STY). For phosphopeptides, quantitation was performed using peptide intensity for modified (STY) P-sites and for proteins using the protein intensity ratio from the protein groups detected in the dimethylated data-set generated by Maxquant (38). Statistical analysis was carried out using Perseus 1.5.0 (39). Intensities were pre-processed by log_2_ transformation and checked for normality. To identify differentially expressed peptides, the Student’s *t*-test were applied to compare groups, *P* values were filtered for the effect of multiple hypothesis testing using the FDR method (<5%). The mass spectrometry proteomics data have been deposited to the ProteomeXchange Consortium (40) via the PRIDE partner repository with the dataset identifier PXD002079.

### Phosphosite localization and kinase assignment

To localize modifications search results were processed using Maxquant that generates a score and probability function to assign confidence to amino acid modification location based on available peak depth present in MS/MS spectra. Upstream kinases were putatively assigned using the NetworKIN algorithm (41) and Phosphosite database (42).

### Viability assays

Cell lines were seeded in 96-well plates at 5000 cells/well in triplicate for each drug treatment and time point. After 2 h, cells were treated with dilutions 5 μM for vemurafenib (PLX4072) alone or in combination with 1.25 or 2.5 μM of the CK2 inhibitor (CX-4945). After 72 h, cell viability for each cell line was assessed by Presto Blue Assay (Life Technologies).

## Results

### Phosphoproteome analysis of *in vitro* drug resistance

**Figure 1:**
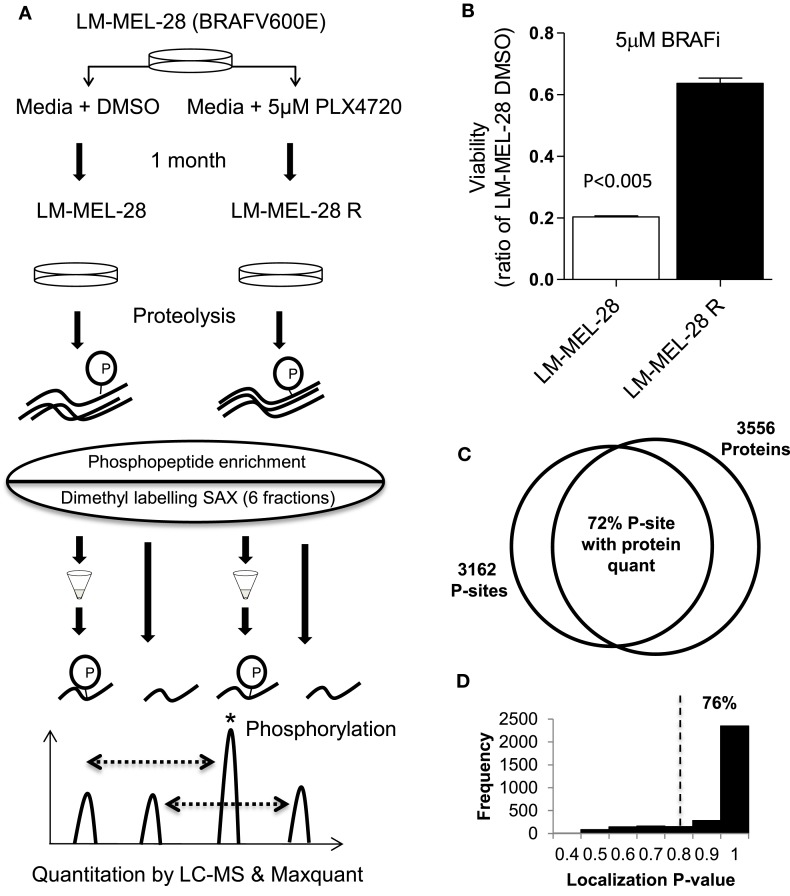
**Phosphoproteomic analysis of *in vitro* drug resistance in melanoma cells**. **(A)** The melanoma cell line LM-MEL-28 *BRAF(V600E)* was exposed to BRAF inhibitor PLX4072 for 30 days to generate LM-MEL28R cell population. Proteins were extracted, digested, and ±TiO_2_ enrichment (for phosphopeptides) or labeled by reductive dimethylation, separated by tip-based strong anion exchange (SAX) chromatography, and analyzed by LC-MS and Maxqaunt. **(B)** The viability of LM-MEL-28R and LM-MEL-28 cells when grown in BRAFi was compared after 3 days (error bars are SD). **(C)** Venn diagram giving the number of P-sites and proteins identified by LC-MS, overlap is calculated where quantitation for P-sites and protein has been determined. **(D)** Histogram of probability values obtained from Maxquant for P-site localization accuracy, dotted line indicates the >0.75 cut-off used for kinase enrichment analysis.

Figure 1A outlines the methodology taken to investigate the phosphoproteome of drug-exposed melanoma cells. To model drug resistance, a *BRAF(V600E)* mutant cell line (LM-MEL-28) was cultured in media containing 5 μM of the selective BRAF inhibitor PLX-4720 for 1 month to generate the stable cell line, LM-MEL-28R. The resistant line LM-MEL-28R was threefold less sensitive to the growth inhibitory effects of PLX4720 than the parental line LM-MEL-28 as shown in a viability assay (Figure 1B). For phosphoproteomic and proteomic analysis, protein extracts were generated, digested with trypsin, and then phosphopeptides were enriched by micro-column based TiO_2_ chromatography analyzed by LC-MS and total peptides labeled by reductive dimethylation using light and heavy isotopes, mixed, separated by SAX chromatography and analyzed by LC-MS; all steps were performed in triplicate. LC-MS identified 3162 unique phosphopeptides (S,T,Y) sequences, mapping to 1164 distinct protein groups and 16,713 none-phosphorylated peptide spectral matches mapping to 3556 protein groups at a FDR of 1% using Maxquant; 836 phosphoproteins (72%) had dual P-site and protein quant estimates providing added confidence in this dataset for detecting changes in phosphorylation occupancy (Figure 1C; Tables S1–S3 in Supplementary Material). The intensity of all phosphopeptides within replicates exhibited a strong positive correlation and low variance (*R*^2^ of 0.75–0.84 and CV 27.26–28.28%). Using a probability function, 76% (2395 of 3162) p[S], p[T], p[Y] sites could be localized with high confidence (>75%) by MS/MS spectra (Figure 1D; Table S3 in Supplementary Material). The intensity of all peptides containing P-sites was used to compare cell populations initially, while Class 1 (>75%) was used for assigning kinase–substrate relationships.

### Phosphorylation of the MAPK1 pathway

**Figure 2:**
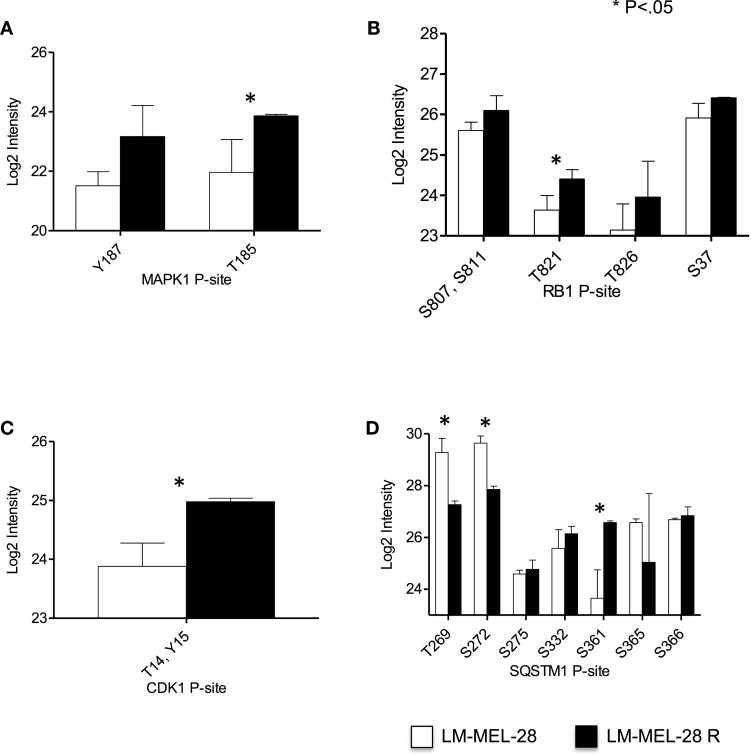
**Phospho-site analysis of MAP kinase pathway output**. **(A–D)** The log_2_ intensity of key P-sites from proteins that function in and downstream of the MAPK01 (ERK1/2) pathway signaling are plotted and analyzed using a Student’s *t*-test (error bars are SD).

Because the MAPK1 pathway is often at the center of acquired drug resistance to BRAFi, we first examined the relative abundance of P-sites in MAPK1, RB1, and CDK1/2, which can provide a measure of MAPK1 signaling (Figures 2A–C). Regulatory P-sites [MAPK1 (T185), RB1 (T821)] and the inhibitory site in CDK1/2 (T/14Y15) were quantified and increased in abundance in LM-MEL-28-R, indicating reactivation/modulation of MAPK1 signaling had taken place in LM-MEL-28R despite continued BRAFi (Figures 2A–C). LC-MS data also provided a site-specific quantitative measure of protein phosphorylation for MAPK1 and RB1 and can thus indicate the activity of the regulatory kinases. To demonstrate this further, we selected the heavily phosphorylated protein, sequestosome-1 (SQSTM1), a known substrate of CDK1 (S269, S272) and demonstrate divergent site-specific protein phosphorylation is detectable. P-site intensity decreased at sites T269, S272 and increased at site S361 providing a snap shot of the activity of multiple kinase and/or phosphatases that target this protein (Figure 2D).

### Proteome of drug resistance

To more accurately determine change in phosphorylation after BRAF drug resistance the proteome of LM-MEL-28 and LM-MEL-28R1 cell populations was compared using isotope coded quantitative proteomics and LC-MS (Table S3 in Supplementary Material). Analysis of these data alone indicated widespread regulation of protein biosynthesis occurs during the development of resistance to BRAFi. Using a twofold cut-off, the majority of proteins were found down-regulated (317) and fewer (151) up-regulated. 1-D gene set enrichment analysis using the log_2_ ratio and the Kyoto Encyclopedia of Genes and Genomes (KEGG, http://www.genome.jp/kegg/) database reflects this and is reported in Table 1 and Table S4 in Supplementary Material). Down-regulated processes indicate major reprograming of metabolic pathways for amino acid metabolism and energy transducing systems (TCA and Glycolysis). Up-regulated processes indicate changes in processes controlling DNA metabolism and the cell cycle.

**Table 1:** **1-D gene enrichment analysis**.

KEGG pathway name	Proteins^a^	Median^b^	Benj. Hoch. FDR
Mismatch repair	14	0.50	3.4E-03
DNA replication	24	0.34	1.3E-04
Nucleotide excision repair	21	0.26	4.6E-04
Cell cycle	40	0.22	3.5E-04
Huntington’s disease	97	−0.37	3.3E-03
Oxidative phosphorylation	73	−0.40	7.2E-04
Alzheimer’s disease	87	−0.41	3.3E-03
Parkinson’s disease	76	−0.41	3.0E-04
Glycolysis/gluconeogenesis	34	−0.48	4.6E-03
Ribosome	72	−0.52	1.2E-08
Cardiac muscle contraction	25	−0.55	6.6E-04
Peroxisome	30	−0.59	2.9E-03
Aminoacyl-tRNA biosynthesis	30	−0.60	3.5E-04
Fatty acid metabolism	24	−0.60	1.6E-03
Citrate cycle (TCA cycle)	27	−0.61	2.2E-03
Valine, leucine, and isoleucine degradation	26	−0.62	4.1E-04
Pyruvate metabolism	27	−0.63	1.4E-03
PPAR signaling pathway	22	−0.67	2.6E-03
Tryptophan metabolism	14	−0.75	3.3E-04

*^a^Number of proteins annotated with the KEGG pathway*.

*^b^Median log_2_ fold change for proteins annotated within the KEGG pathway*.

### Phosphorylation dynamics in drug resistance

**Figure 3:**
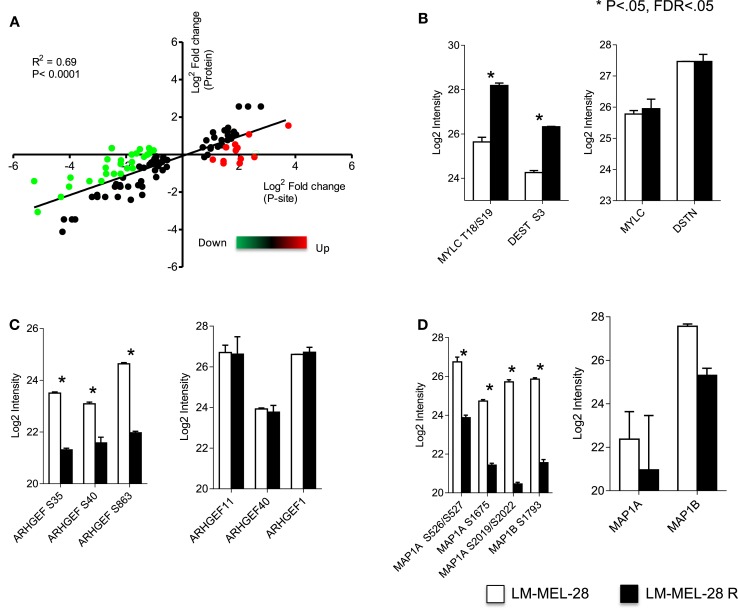
**Phosphorylation rate analysis**. **(A)** Correlation of metrics for P-site effect with protein abundance effect measured between drug sensitive and resistant cell populations. Green = P-site effect twofold > protein effect. Red = P-site effect twofold < protein effect. Log_2_ intensity of P-sites and total protein for key cytoskeletal regulators **(B)** myosin and destrin, **(C)** guanidine exchange factors (11, 40, and 1), and **(D)** microtubule-associated proteins 1A and 1B analyzed using a Student’s *t*-test (error bars are SD).

With estimates of both P-site and protein effect for drug sensitive and resistant populations of LM-MEL-28, we subtracted the protein effect to determine more accurately changes in the rate of phosphorylation at significant sites. Protein quantitative estimates for 2895 sites (~72%) were available and using the following equation (Phospho rate = log_2_ Phospho − log_2_ Protein) specific post-translational activity (kinase or phosphatase) was inferred. Figure 3 is an *x*/*y* scatter plot of the significant P-sites (*n* = 148, Student’s *t*-test FDR corrected *P* < 0.05) where *x* is log_2_ P-site ratio (R1/S1) and *y* is log_2_ protein ratio (R1/S1). Data were well correlated indicating a large effect of protein abundance on P-site abundance (Pearson’s *R*^2^ = 0.6, *P* < 0.0001) (Figure 3A). Forty-seven accurately localized (*P* < 0.75) P-sites were differentially regulated by a minimum of twofold after protein abundance was subtracted, and these were selected for kinome analysis using Phosphosite.org and NetworKIN databases (41, 42) (Table 2). Seventeen sites originated from singly phosphorylated peptides, 30 sites were from 15 doubly phosphorylated peptides, of which 5 had a second site where the P-site localization was ambiguous (Class 2) (Table S5 in Supplementary Material). All accurately localized sites (Class 1) were used for further analysis, and it was accepted that for sites originating from the same peptide the quantitative value would amount to the sum of regulation at each site.

**Table 2:** **Regulated phosphosites in drug resistant cells and the prediction of putative regulatory kinases**.

Protein names	Gene names	P-site	Diff^a^	*R*	PhosphoSitePlus kinase	NetworKIN
Protein kinase C alpha type	PRKCA	T497	2.21	+		PDHK1
Serine/threonine-protein phosphatase 2A regulatory subunit B” subunit alpha	PPP2R3A	S692	1.41			MAPK3, MAPK1, CDK1
Myosin regulatory light chain 12B	MYL12B	S25	2.74	+	ILK; DLK; DAPK1; ROCK1; AurB; smMLCK; DAPK3; CAMK1A; CRIK; MRCKA; PKCA; PAK1	
Myosin regulatory light chain 12B	MYL12B	T24	2.74	+	ILK; DLK; ROCK1; smMLCK; DAPK3; CRIK	ROCK2
G-protein coupled receptor 143	GPR143	S343	1.28			
Destrin	DSTN	S3	2.29	+	LIMK2; LIMK1; TESK1	
40S ribosomal protein S6	RPS6	S236	2.49	+	PKCD; p90RSK; p70S6K; RSK2	p70S6K
Nucleoprotein TPR	TPR	S2155	1.59			MAPK1
Choline-phosphate cytidylyltransferase A	PCYT1A	S347	1.66			
E3 ubiquitin-protein ligase HERC2	HERC2	S2928	1.32			CK2alpha
Choline-phosphate cytidylyltransferase A	PCYT1A	S343	1.59			
DNA replication licensing factor MCM3	MCM3	S756	1.16			CK2alpha
DNA replication licensing factor MCM3	MCM3	S717	1.15			CK2alpha
60S acidic ribosomal protein P1	RPLP1	S104	1.92			CK2alpha
60S acidic ribosomal protein P2	RPLP2	S105	1.80			GRK2, CK2alpha
Insulin receptor substrate 2	IRS2	S736	1.34			GSK3alpha, GSK3beta
Choline-phosphate cytidylyltransferase A	PCYT1A	S331	−1.25			
ATP-dependent RNA helicase DDX24	DDX24	S82	−1.31		Chk1	
Ras-related GTP-binding protein C	RRAGC	S95	−1.25			
Ankyrin repeat and SAM domain-containing protein 1A	ANKS1A	S663	−1.53			
Septin-9	SEPT09	S85	−1.24			CK1delta
CLIP-associating protein 1	CLASP1	S415	−1.75			NEK2, CaMKIIalpha
C-Jun-amino-terminal kinase-interacting protein 4	SPAG9	S730	−1.25			
C-Jun-amino-terminal kinase-interacting protein 4	SPAG9	S733	−1.25			
Rho guanine nucleotide exchange factor 40	ARHGEF40	S262	−1.80			
MAP7 domain-containing protein 1	MAP7D1	S113	−1.69			
Niban-like protein 1	FAM129B	S646	−1.04			
Sequestosome-1	SQSTM1	S272	−1.40	+	CDK1	MAPK3
60S ribosomal export protein NMD3	NMD3	T470	−1.81			
Niban-like protein 1	FAM129B	S641	−1.23			
Sequestosome-1	SQSTM1	T269	−1.62	+	CDK1	MAPK3
Syntaxin-12	STX12	S142	−1.87			
E3 ubiquitin-protein ligase	NEDD4L	S308	−1.06			PDHK1, GSK3beta
Rho guanine nucleotide exchange factor 11	ARHGEF11	S35	−1.70			PAK4
E3 ubiquitin-protein ligase	NEDD4L	S307	−1.30	+	PKACA; SGK1	TGFbR2
Rho guanine nucleotide exchange factor 1	ARHGEF1	S919	−2.42			
Microtubule-associated protein 1A	MAP1A	S764	−1.46			
Microtubule-associated protein 1A	MAP1A	S765	−1.46			
Microtubule-associated protein 1A	MAP1A	S1913	−1.89			
Cation-independent mannose-6-phosphate receptor	IGF2R	S2484	−2.74		CK2A1	CK2alpha
Nestin	NES	S680	−2.31			CDK1, CDK5
Microtubule-associated protein 1B	MAP1B	S1793	−2.05			GSK3beta
Microtubule-associated protein 1B	MAP1B	S1797	−2.05			GSK3beta
PDZ and LIM domain protein 4	PDLIM4	S112	−2.08			
Microtubule-associated protein 1A	MAP1A	S2257	−3.85			CK1alpha, CK1delta
Microtubule-associated protein 1A	MAP1A	S2260	−3.85			CK1alpha, CK1delta

*^a^Log_2_ fold change corrected for protein effect (see text), R (known regulatory site)*.

### Kinase enrichment analysis

Using the phosphosite.org and NetworKIN databases, regulatory kinases for 29/46 P-sites could be assigned and are reported in Table 2 and Tables S3 and S5 in Supplementary Material. Of the 46 P-sites, 11 were in key cytoskeletal regulators and kinase predictions were available for 7 of these sites. For example, myosin regulatory light chain, MLC12A/B/9 (T18 and S19) destrin/cofilin (S3) predicts the activity of ROCK1 and LIMK1/2 protein kinases (Figure 3B). P-sites in three distinct guanidine exchange factors (GEF’s 11, 40, and 1) were also regulated by phosphorylation and S35 in GEF40 is putative substrate for the p21-associated kinase PAK4 (Figure 3C; Table 2). Phosphorylation of two microtubule-associated proteins (MAPs) increased at six sites (Figure 3D). Here, sites S2019, S2022 predicted the activity of CK1A on MAP1A and S1793, S1797 are putative substrates for GSK3β on MAP1B. Other sites of note for which kinase–substrate predictions were determined included two MAPK1 substrates TPR (S2155) and PPP2R2A (S692). Sites in the key signaling molecules insulin receptor substrate 1 (IRS1, S736), the insulin-like growth factor 2 receptor/cation-independent mannose-6-phosphate receptor (IGF2R/CI-MPR, S2484), and protein kinase C (PKC, S497) indicated activity of GSK3α/β, CK2A, and PDHK1, respectively. Nestin (S680) and sequestosome-1 (T269, S272) decreased in phosphorylation and are predicted to be substrates of CDK1, consistent with the increase in inhibitory phosphorylation of CDK1 (Y15/T14) measured. Finally, Casein kinase 2 alpha (CK2A) was predicted to regulate six sites, four of which increased in abundance for proteins that function in core processes of DNA replication and damage responses [MCM3 (S711, S672) and HERC2 S2928] and protein translation (RPLP1/2, S104/105) (Table 2).

### Meta-analysis

Recently, Girotti et al. identified major regulation of phosphoproteins involved in cytoskeletal and cell invasion gene ontology and interaction modules occurs in melanoma cells with acquired BRAFi resistant *in vitro* (32). To investigate our results in the context of this and other data-sets a meta-analysis of datasets including Girotti et al. and Old et al. (a measure of short-term BRAFi in melanoma) was completed Table S7 in Supplementary Material (31, 32). Several sites in cytoskeletal proteins [e.g., Nestin (S680/768), Cortactin (S405) MAPB1 (S1793)] were commonly regulated in both our and the Girotti et al. (32) datasets. Less overlap is observed with the Old et al. screen, with only Cortactin (S405/S401) and NES (S768) regulated in all three data-sets. Additionally, we compared our data to an shRNA screen by Sun et al. for factors that influence the expression of EGFR in acquired drug resistance (13). Both SOX-10 and MTA2 were identified in the screen and both are measured in our proteomic data. We observed no change in SOX-10 protein expression, but an increase in MTA2 expression in drug resistance cells (Figure S1 in Supplementary Material).

### Drug resistant cells are sensitive to CK2 co-inhibition

**Figure 4:**
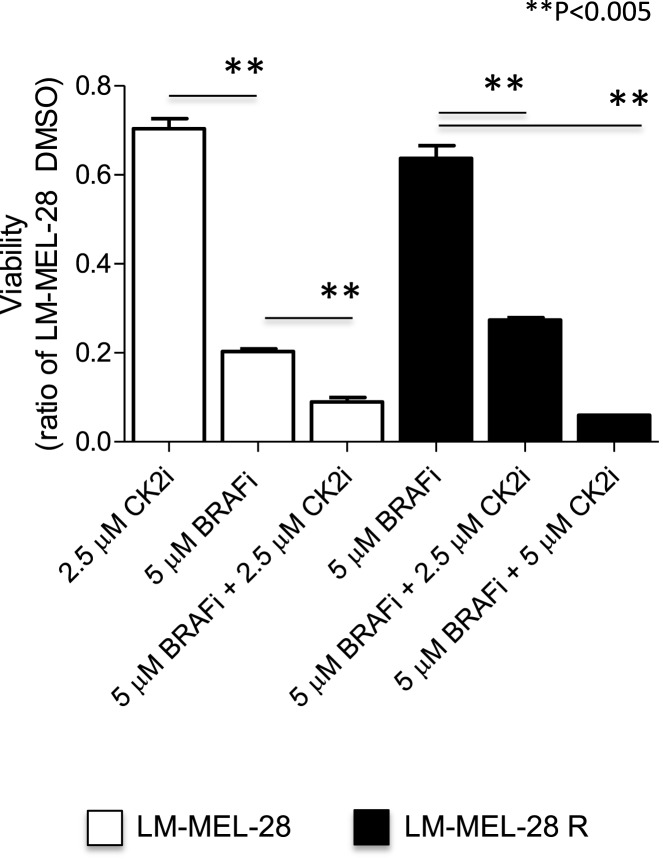
**CK2i of the drug resistant cell line (LM-MEL-28-R1)**. The effects of co-inhibition with the CK2 inhibitor CX-4945 and BRAFi (PLX4072) on proliferation of the drug resistant cell population LM-MEL-28-R1 were quantitated by viability assay, analyzed using a Student’s *t*-test (error bars are SD).

Based on the measured increase in phosphorylation of several putative CK2 sites in LM-MEL-28R and our previous finding that CK2 inhibition is synergistic with BRAF inhibition in *BRAF(V600E)* mutated cells (43), we tested whether LM-MEL-28R was sensitive to CK2 inhibition. Figure 4 demonstrates that the resistant line was sensitive to co-inhibition with CK2 inhibitor CX-4595 and that this inhibition was beyond what was observed for CK2 alone in the drug sensitive LM-MEL-28. A quantitative reduction in cell growth over several concentrations of inhibitor (<50% at 2.5 μM and <90% at 5 μM) was observed in LM-MEL-28R.

## Discussion

Changes in the phosphoproteome of a *BRAF(V600E)* mutant melanoma cell line that occur after the development of drug (PLX4720) resistance *in vitro* are described here. Using a single step phosphopeptide enrichment followed by LC-MS analysis and label-free quantitation using the freeware Maxquant, we accurately detected and measured ~2700 phosphorylation events and 3556 proteins. Initially, we quantitated the viability of both unexposed and drug resistant populations in the presence of BRAFi. We observed that although viability was reduced after drug adaption, stable growth was maintained and cells were able to propagate in the presence of 5 μM PLX4720. Our *in vitro* system provided a suitable model to measure phosphorylation in drug resistance *in vitro*; and through kinases landscape analysis the activity of several kinases regulating these events was predicted.

**Figure 5:**
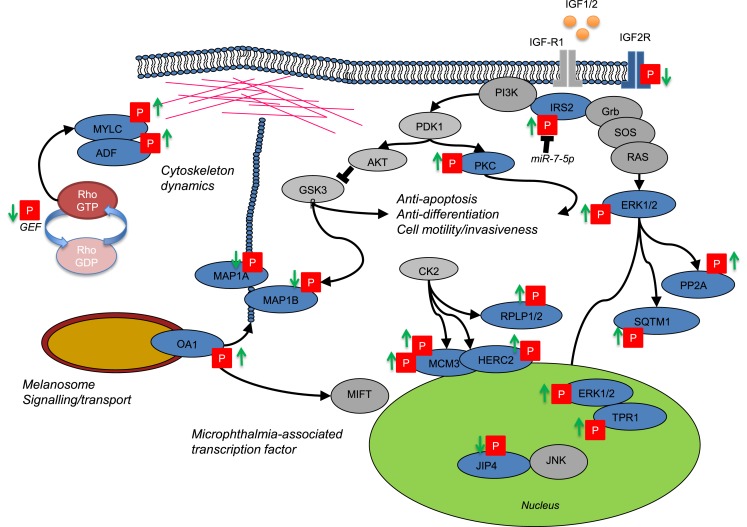
**Network cartoon summarizing the prominent differences in phosphorylation measured between BRAFi sensitive and resistant cell populations**.

Drug resistance in melanoma often occurs through reactivation of MAPK signaling despite continued exposure to the inhibitor (10). Through selective analysis of regulatory P-sites in the MAPK1 signaling pathway (ERK1/2) and downstream cell cycle regulators (RB1 and CDK1), our cell model was consistent with MAPK reactivation in resistant cells despite exposure to BRAFi. With this data set, we next investigated the relationship between protein phosphorylation and protein abundance. A measure for protein abundance was available for ~72% of measured P-sites. The measure for protein was based upon all identified unmodified peptides mapping to the same protein group as the P-site-derived peptide spectral match. This measure could account for the dominant effects of protein metabolism (synthesis and degradation) often observed in cells during long-term adaptive responses. As expected, the abundance of the majority of P-sites closely followed that of protein expression, with only a small subset (46 P-sites) exhibiting changes in abundance that could not be accounted for by changes in the rate of protein turnover. The most likely explanation for this divergence is the activity of kinase(s) or phosphatase(s) with specific regulatory functions in cellular adaption to BRAF inhibition. Focusing on kinases where the most probable enzyme–substrate relationship(s) can be mapped, potential regulatory mechanisms were identified and are discussed below and summarized in Figure 5.

### Drug resistance induces de/phosphorylation of the cytoskeleton regulators

Cytoskeletal changes are central to the phenotypic transitions that occur in tumor progression, altering invasiveness, metastasis, and resistance to therapy and this was reflected in our data. P-sites that are key regulatory residues in proteins controlling both actin and microtubule-based filaments were altered beyond protein metabolic control. Destrin (actin-depolymerization factor, ADF) is responsible for actin stability (44) and Ser-3 can be phosphorylated by LIM domain kinase 1 and 2 (LIMK1/2) to induce cytoskeletal reorganization to form stress fibers, membrane blebs and alter cell adhesion through the formation of F-actin in non-muscle tissue (45, 46). Myosin light chain (12A/B/9) phosphorylation of S19 and T18 provide evidence for increased activity of several up-stream kinases [myosin light chain kinase (MLCK), Rho-associated kinase (ROCK), citron kinase, leucine zipper interacting kinase ZIPK/DAPK3, and CDC42 binding kinase]. Functionally, phosphorylation of S19/T18 alleviates auto-inhibition of the MYLC globular heads and promotes interaction with actin to form bipolar filaments (47). The activating signals for this are diverse, ROCK2 is activated by the small GTP-binding protein RhoA, which is dependent on the activity of GEFs (48, 49). In the resistant cell population, a drop in phosphorylation of three different GEF’s, including S35 in GEF11 was measured. Phosphorylation of GEF11 by the Cdc42 effector kinase PAK4 and p38 MAPK both lead to a drop in GEF activity (50, 51). Kinase enrichment analysis predicted a C-terminal site S35 to be a target for PAK4, indicating a novel site where PAK4 may regulate GEF activity in drug resistant cells. In the microtubule-based cytoskeleton, reduced phosphorylation of MAPs indicated altered tubule stabilization and several sites near microtubule binding domains could influence the tethering of cargo for transportation (52). P-site S1793 and S1797 in MAP2A are putative sites for GSK3-β. GSK3-β activity couples extra-cellular matrix (ECM) signaling to the actin/microtubule cytoskeleton during cell migration (53).

The regulation of cytoskeletal dynamics by Rho/ROCK and GEF signaling is a key driver of the phenotypic transitions or switching that can change the migratory phenotype of cells (54). In melanoma, transcriptional networks that have roles in mesenchymal and amoeboid transitions alter during metastasis and response to therapy (55). These changes may contribute to the intrinsic invasive phenotypes that have been observed in response to inhibitor therapy (25, 32). ROCK1 and 2 promote myosin phosphorylation and actin fiber formation to drive amoeboid movement, where cell membranes undergo extensive blebbing allowing cells to deform and pass through voids in the surrounding matrix (56). Co-inhibition of ROCK signaling has recently been shown to enhance the anti-proliferative effect of the BRAFi PLX4720 and supports our observation that drug resistant populations utilize ROCK signaling as a pro-survival mechanism (57). Through a meta-analysis of our results with other drug-exposed cell models (31, 32), a clear functional role can now be confirmed for the phosphorylation of proteins that function in the cytoskeleton. However, this analysis revealed that only 20/145 P-sites were shared between our data and that of Girotti et al. (32). This discrepancy could be explained by the myriad of possible mechanisms that can mediate drug resistance and is likely to depend on tumor genotype, heterogeneity, and locale; where each generates a unique cytoskeletal organization of maximum fitness. Outside of biological variation, technical differences in data generation and analysis may underlie the inconsistency in the P-sites identified. However, in Girotti et al., despite differences in methodology and only a twofold cut-off being applied to assign significance, of the sites that do overlap the majority of P-sites (13) exhibit a similar direction of regulation. While carrying out this meta-analysis, we also compared the proteomic data to the results of an shRNA screen for mechanisms of EGFR-based drug resistance in melanoma. Here, we identified MTA2 but not SOX-10 protein expression as altered in drug resistant cells. In Sun et al. (13), MTA2 is ruled out as a false-positive mediator by a targeted approach. These data provide an indication that LM-MEL-28, does not acquire BRAFi resistant through expression of EGFR receptor via SOX-10 attenuation; and could further explain the discrepancy in regulated P-sites with Girotti et al., where EGFR signaling is required for growth of the resistant cell population (32).

### Melanosome signaling through G-protein couple receptor-143 (OA1)

G-protein-coupled receptors can generate signals key to the development of resistance to BRAF inhibitor therapy (58). Here, we identified novel sites of phosphorylation (S331/S343) in the GPCR 143, also known as ocular albinism type 1 (GPR143/OA1), that increased in BRAFi resistant cells (Table 1; Figure 5). OA1 is a pigment-cell specific G-protein receptor for tyrosine, l-DOPA, and dopamine it also localizes to intracellular melanosomes and forms a key component of melanosome biogenesis and transport (59–61). OA1 regulates expression of the MITF, sustaining its expression and promoting melanocyte differentiation (62). Oncogenic BRAF can suppress MITF expression preventing normal melanocyte differentiation and promoting transformation to a de-differentiated proliferative state (63). In GPR143/OA1, S331/S343 reside in the C-terminal cytoplasmic domain and while no kinase prediction was assigned, phosphorylation here could drive the classical recruitment of beta-arrestins and lead to inactivation of G-protein signaling by OA1 leading to further de-differentiation observed in drug resistant tumors (64). OA1 also signals through the actin/microtubule cytoskeleton to regulate the transport of melanosomes from the perinuclear region to the cell periphery and could in-part drive phosphorylation dynamics of the cytoskeleton indicated above (59). Finally, S331 is directly adjacent to a two amino acid “WE” domain vital for the correct localization of OA1 protein to the melanosome (65). Mutation of WE > AA redirects OA1 to the plasma membrane (65). The role of OA1 phosphorylation in protein localization, melanosome and cytoskeleton signaling and how this facilitates drug resistance remain to be tested.

### Key P-sites in known signaling nodes reflect MAPK1 reactivation

T497 in protein kinase C alpha (PKCα) increased in expression and phosphorylation in drug resistant cells. T497 in PKCα is located in the activation loop and phosphorylation is essential for full catalytic activity of PKCα (66). Phosphorylation of T497 by PDPK1 (PDK1) is classically dependent on phosphatidylinositol metabolism and PI3K activation induced by GPCR or TRK signaling (Figure 5). PKCα activity in melanoma is highly context dependent with roles in both oncogenesis and growth suppression (67). PKCα can contribute to activation of the MAPK pathway through direct phosphorylation of RAF substrates to activate ERK, or promote the c-Jun N-terminal kinase (JNK) MAP kinase pathway through association with RACK1 (68, 69). RACK1 shuttles PKCα to target the stress-related MAPK JNK for phosphorylation leading to constitutive activation of p38 MAPK signaling (69). Interestingly, we measured dephosphorylation of S730/733 in SPAG9 (JIP4), a scaffold protein involved in the spatial organization of MAP kinases and a mediator of c-Jun N-terminal kinase. The meta-analysis of Girotti et al. (32) supported this finding and while kinases/phosphatases able to regulate SPAG9 S730/733 phosphorylation remain unreported our data indicates that regulation may be key to the rewiring of MAPK signaling in cells adapted to BRAFi. A further mechanism able to reactivate ERK1/2 signaling in BRAFi resistance was indicated by the increased phosphorylation of the ERK/1/2 substrate TPR at S2155 (Table 1). TPR is a nuclear pore complex protein and chromatin regulator that in response to ERK1/2 phosphorylation can bind and localize ERK1/2 to chromatin (70). During short-term exposure to BRAF and MEK inhibitors phosphorylation of TPR at S2155 reduces in *BRAF(V600E)* mutant cell lines (43). The recovery of TPR phosphorylation in the face of chronic BRAFi appears to be associated with the re-establishment of MAPK nuclear signaling in drug adapted cells.

Evidence for potential upstream mechanisms for ERK reactivation is provided by a change in phosphorylation of insulin receptor substrate (IRS2), a downstream effector of insulin-like growth factor receptor 1 (IGF-1R). IGF-1R signaling in cancer cells results from up-regulation of the receptor or its ligands (IGF-I and IGF-II) and contributes to the emergence of chemotherapeutic resistance. Insulin receptor substrate (IRS1/2) proteins transmit oncogenic signals through PI3K and ERK signaling modules (Figure 5). IRS1/2 also mediate the termination of IGF-IR signaling and resistance to PI3K inhibitors occurs through a reduction in this feedback inhibition [reviewed in Ref. (71)]. We measured phosphorylation of IRS2 at two sites, (i) S736 confidently localized and predicted to be regulated by GSK-3α/β and (ii) an ambiguous P-site (either S730/731/735/740 or Y742) in the same peptide. A lack of clarity for the position of the second site makes it difficult to predict, which kinase(s) may be responsible for the regulation that we observed. However, phosphorylation of IRS2 represents a key signaling process where cells become reprogramed through PI3K to activate PDK1-PKC/PKB(AKT) or through GRB2-SOS to activate the Ras-MAPK pathway directly (Figure 5) (72). IGF-1R has been shown to be up-regulated in drug resistant melanoma cell lines previously (12). Recently, IRS2 was also found up-regulated in BRAFi (PLX4032) resistant tumors and blocking or eliminating IRS or subsequent PI3K-mediated signaling may provide therapeutic potential (12, 73). More specifically, IRS-2 is a target of miR-7-5p found down-regulated in melanoma (74). miR-7-5p down-regulation is associated with increased cell migration and metastasis, and using RNA interference (RNAi) IRS-2 was shown to regulate this phenotype through the PKB/AKT signaling node (74, 75). In support of a role for IGF signaling, a decrease in the phosphorylation of a CK2 site (S2484) in the cytoplasmic domain of the insulin-like growth factor receptor II (IGFR2) known also as the CI-MPR receptor was detected in BRAFi drug resistant cells. This protein acts as both the receptor for IGF2 and mannose-6-phosphate and is implicated in both G-protein signaling and the targeting of lysosomal enzymes. In CHO cells, phosphorylation of this site regulates changes in the trafficking of the receptor in the Golgi-network (76) and down-regulation of plasma membrane IGFR2 is associated with increased signaling through IGF-R1 (77).

This study demonstrated a simple and effective approach to detect kinase activity important in the transition of cells from a BRAF sensitive to BRAF resistant phenotype. Once detected these kinase present themselves as potential targets for future

co-therapies. During our analysis, we detected increases in the phosphorylation and abundance of proteins involved in processes related to DNA metabolism. Several of these sites were substrates for CK2A, and we tested if long-term exposure to BRAFi provided protection from the synergistic inhibitory effects of protein kinase CK2A–BRAF co-inhibition previously observed in *BRAF(V600E)* mutant melanoma (43). This was not the case with an additive effect (>50%) being observed in both parental and resistant populations, suggesting that this drug combination could be effective in reducing the emergence of resistant cell populations. We have previously demonstrated that CK2 plays an important role in priming the activity of Akt through phosphorylation at S129, and that controlling CK2 activity is an effective strategy in preventing cell growth in BRAF melanoma and BRAF thyroid carcinoma (43). Notwithstanding the importance of Akt-driven growth pathway, CK2 is a ubiquitous serine/threonine kinase and in the nucleus plays an important role in modulating DNA-damage and repair machinery (78, 79); it is likely that the inhibitory effect of blocking CK2 leads to wide-spread modulation in numerous other pathways that support cell proliferation. Understanding the mechanistic significance of how CK2 regulates these other pathways in melanoma needs ongoing research.

## Conclusion

A central paradigm of acquired drug resistance in BRAF mutant melanomas is the reactivation of MAPK signaling (10). In this work, a quantitative MS method measuring both the phosphoproteome and proteome was developed and implemented to describe novel phosphorylation-based signaling events in cells after this transition *in vitro*. We identified increased MAPK01 phosphorylation alongside well-known and novel protein phosphorylation events driven by this and other kinases. Regulation of key substrates in Rho/ROCK signaling axis provided evidence for cytoskeletal rearrangements able to facilitate a phenotypic switch in cell motility that evolve during BRAFi therapy. Importantly, our study provided evidence for signaling events in several proteins (IGFR2, IRS1, PKC, and GEFs) associated with established pathways of drug resistance in melanoma and other cancers (12, 80). Phosphorylation of IRS1 re-enforces the importance of IGF signaling in drug resistant melanoma as a valid target for co-therapy. Novel sites identified indicate new and untested mechanisms able to promote cell survival and these require confirmation *in vivo*. The diversity of drug resistance mechanisms discovered in melanoma so far indicates a need to develop an individualized approach to multi-targeted cancer treatment. The MS-driven phosphoproteomic method described here can be readily applied to the analysis of tumors biopsied before, during, and after treatment to provide a direct readout for kinases that are drug-able targets in relapsed patients.

## Conflict of Interest Statement

The authors declare that the research was conducted in the absence of any commercial or financial relationships that could be construed as a potential conflict of interest.

## Supplementary Material

The Supplementary Material for this article can be found online at http://journal.frontiersin.org/article/10.3389/fonc.2015.00095
