# Supplementary figures and images for: Phosphoproteomic Analysis of Cell-Based Resistance to BRAF Inhibitor Therapy in Melanoma
Source: Front Oncol. 2015 May 15;5:95. doi: 10.3389/fonc.2015.00095 (PMC4432663; doi:10.3389/fonc.2015.00095)

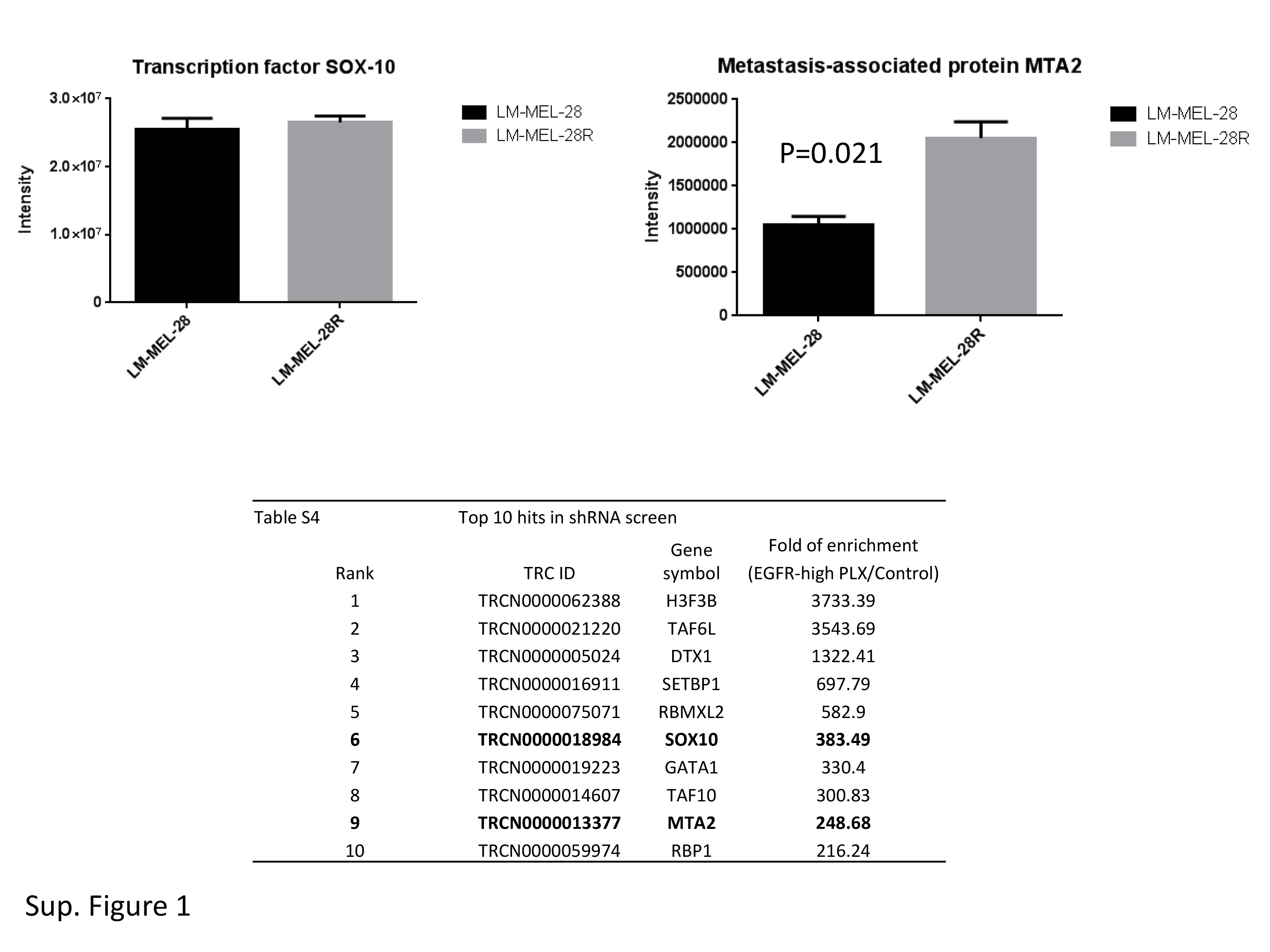

Supplement: Supplementary file 2 [file image_1.tif]
